# Distributed Temporal Coding of Visual Memory Categories in Human Hippocampal Neurons

**DOI:** 10.21203/rs.3.rs-5486087/v1

**Published:** 2024-11-26

**Authors:** Xiwei She, Bryan J. Moore, Brent M. Roeder, George Nune, Brian S. Robinson, Brian Lee, Susan Shaw, Hui Gong, Christianne N. Heck, Gautam Popli, Daniel E. Couture, Adrian W. Laxton, Vasilis Z. Marmarelis, Samuel A. Deadwyler, Charles Liu, Theodore W. Berger, Robert E. Hampson, Dong Song

**Affiliations:** 1 Department of Biomedical Engineering, Viterbi School of Engineering, University of Southern California; 2 Wake Forest Institute for Regenerative Medicine, Wake Forest University School of Medicine; 3 Department of Neurological Surgery, Keck School of Medicine, University of Southern California; 4 Department of Neurology, Rancho Los Amigos National Rehabilitation Hospital; 5 Department of Neurology, Wake Forest University School of Medicine; 6 Department of Neurosurgery, Wake Forest University School of Medicine

**Keywords:** Human hippocampus, neurons, spike, memory category, spatio-temporal code, memory decoding model

## Abstract

The hippocampus is crucial for forming new episodic memories. While the encoding of spatial and temporal information (where and when) in the hippocampus is well understood, the encoding of objects (what) remains less clear due to the high dimensions of object space. Rather than encoding each individual object separately, the hippocampus may instead encode categories of objects to reduce this dimensionality. In this study, we developed and applied a combined experimental-modeling approach to investigate how the hippocampus encodes visual memory categories in humans. We recorded spikes from hippocampal CA3 and CA1 neurons in 24 epilepsy patients performing a visual delayed match-to-sample (DMS) task involving five image categories. An ensemble multi-temporal-resolution classification model was employed to decode these visual memory categories from the hippocampal spiking activity with moderate numbers of trials. This model enables the identification of the spatio-temporal characteristics of hippocampal encoding through its interpretable representations. Using this model, we estimated the optimal temporal resolutions for decoding each visual memory category for each neuron in the ensemble. Results indicate that visual memory categories can be decoded from hippocampal spike patterns despite the short data length, supporting the presence of category-specific coding in the human hippocampus. We found that hippocampal neuron ensembles encode visual memory categories in a distributed manner, akin to a population code, while individual neurons use a temporal code. Additionally, CA3 and CA1 neurons exhibit similar and redundant information regarding visual memory categories, likely due to the strong and diffuse feedforward synaptic connections from the CA3 region to the CA1 region.

## Introduction

The hippocampus is a brain region critical for the formation of new episodic memories^1–3^. Impairment to the hippocampus due to diseases or injuries leads to profound memory deficit^4–7^. Therefore, hippocampal neurons are naturally positioned to encode episodic memory-related information such as what (object), when (time), and where (space) of past events. While, due to their relatively low dimensionalities, how hippocampal neurons encode spatial (where) and temporal (when) information is well characterized^8–11^, how different objects (what) are encoded in the hippocampus is much less understood.

Indeed, the space of objects has nearly infinite dimensions. It is infeasible for the hippocampus to encode every individual object separately. One possible strategy is to encode categories and/or features of objects to reduce the dimensionality of the object space. This hypothesis is supported by nonhuman primate (NHP) studies, which showed that some hippocampal neurons increased their firing rates in response to visual stimuli (images) within the same categories during a memory task^12^. Moreover, such category-specific responses in the hippocampus to visual stimuli were also observed in humans^13^.

Previous studies have primarily investigated hippocampal encoding of objects at the single-neuron level by analyzing averaged activity patterns^12–16^. In these studies, perievent histograms of neurons are calculated from spike patterns across many trials using a preselected bin size (temporal resolution of encoding). Category-specific neurons were then identified based on the correlation between their perievent histograms and image categories. Although this approach has provided valuable insights into hippocampal neuronal encoding, the precise nature by which the hippocampus neuronal ensemble encodes category-specific information through its spatio-temporal patterns of spikes is not fully characterized. For example, it is unclear how the same hippocampal neuron encodes multiple categories or whether hippocampal neurons encode categories using the same or different temporal resolutions.

**Figure 1:**
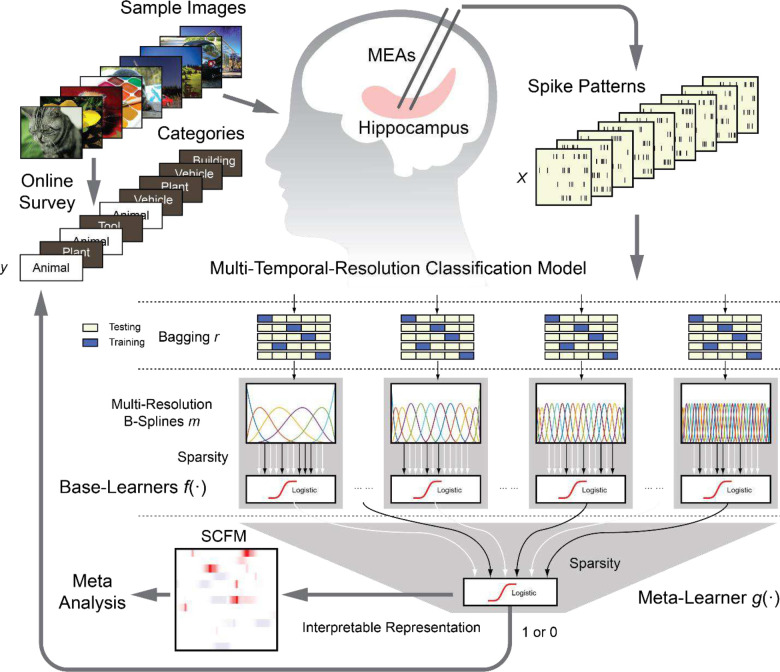
Decoding visual memory categories from spatio-temporal patterns of spikes recorded in the human hippocampus using an ensemble multi-temporal-resolution classification model. This model provides interpretable model representations of spatio-temporal characteristics of spike patterns for encoding specific memory categories.

To answer these questions, we developed and applied a combined experimental-modeling approach to quantitatively investigate hippocampal encoding of visual memory categories in human subjects. Hippocampal CA3 and CA1 spikes were recorded from epilepsy patients performing a visual delayed match-to-sample (DMS) task involving multiple categories of images. An ensemble multi-temporal-resolution classification model was used to decode visual memory categories from the hippocampal spiking activities with a relatively small number of trials (Fig. 1). This model directly identifies the spatio-temporal characteristics of the hippocampal encoding with its interpretable model representations. Utilizing an ensemble learning strategy, optimal temporal resolutions of decoding were estimated for each neuron in the neuronal ensemble for each visual memory category. Results show that visual memory categories can be decoded from hippocampal spike patterns with significant accuracies despite the short data length (small number of trials), which strongly supports the existence of category-specific coding in the human hippocampus. Hippocampal neuron ensembles encode visual memory categories in a distributed manner, similar to a population code, while each individual neuron encodes visual memory categories with a temporal code. In addition, hippocampal CA3 and CA1 neurons contain similar and redundant information of visual memory categories, possibly due to the strong feedforward synaptic pathway between the two regions^17^.

## Results

### Human Subjects

Twenty-four patients with medically refractory focal epilepsy and mild-to-moderate memory abnormalities were enrolled in the study. The age of subjects ranged from 20 to 62 years, with an average age of 36.6 ± 12.2 years. The subjects comprised 13 males and 11 females, ensuring a balanced gender distribution. Among the 24 subjects, 11 had bilateral recordings on both the anterior and posterior regions of the hippocampus; 8 had bilateral recordings on the anterior region of the hippocampus; 5 had unilateral recordings on the anterior region of the hippocampus. The average number of recorded neurons was 30.0 ± 15.7 per subject. During the memory tasks, subjects completed an average of 149.5 ± 25.4 trials.

### Decoding Memory Categories Using a Multi-Temporal-Resolution Classification Model

**Figure 2:**
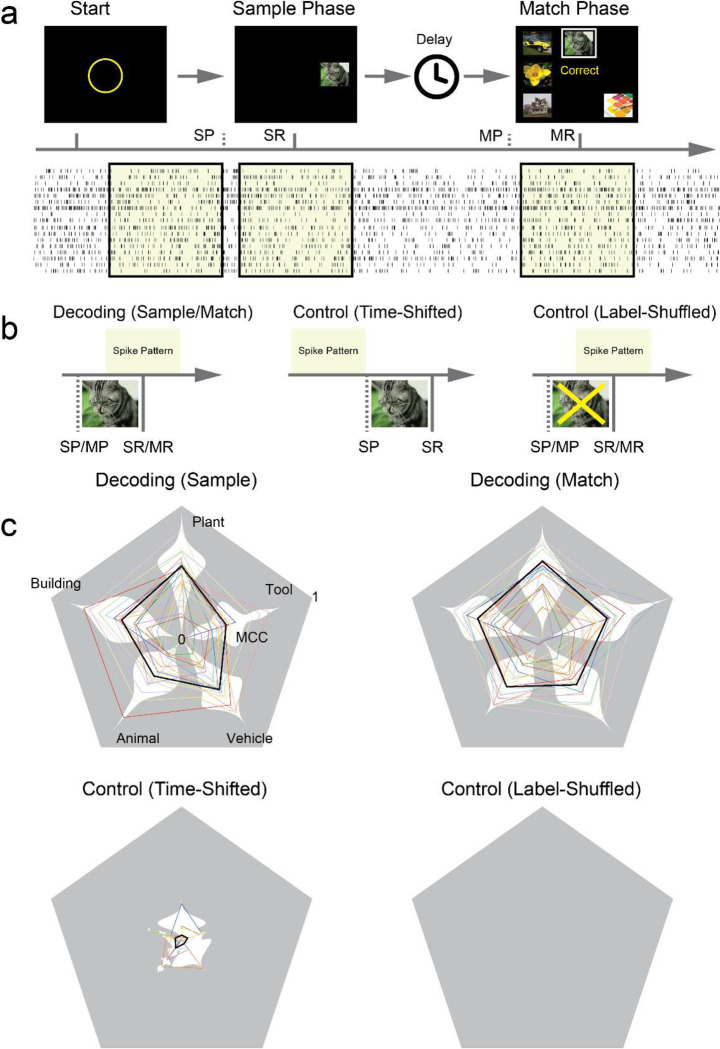
Categories of sample images can be decoded from spatio-temporal patterns of spikes recorded during Sample Response and Match Response events of a delayed DMS task in human subjects (n=24). **a**: DMS task paradigm. SP: Sample Presentation; SR: Sample Response; MP: Match Presentation; MR: Match Response. **b**: decoding cases and control cases in the modeling. **c:** decoding performance of the five memory categories. Color lines: MCCs of individual subjects; Black thick lines: average MCCs across all subjects; White shades: distributions of MCCs within categories.

We apply a multi-temporal-resolution classification model^18^ to test whether hippocampal spiking activities contain visual memory category-specific information (see “Methods”). This model decodes memory categories (outputs) from hippocampal spatio-temporal patterns of spikes (inputs) recorded at different phases of the memory-dependent DMS task. Two decoding cases and two negative control cases are included (Fig. 2a, b).

In the two decoding cases, spatio-temporal patterns of spikes during “Sample Response” (memory encoding) events and “Match Response” (memory retrieval) events are used as model inputs, respectively. Binary (1 or 0) labels of the five memory categories of the sample images, i.e., “Animal,” “Building,” “Plant,” “Tool,” and “Vehicle”, were used as model outputs. The objective of these two cases was to assess the model’s ability to decode memory categories when memory was being encoded and retrieved, respectively.

In the first control case (Time-Shifted), the time windows of spike patterns are shifted to be before the “Sample Presentation” events, so the input spike patterns contain no information about the output memory categories. In the second control case (Label-Shuffled), model outputs are randomly shuffled across samples, thereby disrupting any potential correlation between them and the input spike patterns. These two negative control cases are included to ensure that the model does not overfit the data so the decoding results from the decoding cases are reliable.

Results show that in both “Sample Response” and “Match Response” cases, the model yields significant classification accuracies, indicated by the Matthews correlation coefficients (MCCs) between true labels and predicted labels, in most categories and subjects (Fig. 2c, top). Average MCCs of the “Sample Response” case are 0.29 ± 0.16, 0.39 ± 0.23, 0.47 ± 0.19, 0.29 ± 0.16, 0.40 ± 0.19 for the five memory categories, respectively (Supplementary Table 1; note the chance-level MCC is equal to 0). In the “Match Response” case, MCCs of the five memory categories are 0.38 ± 0.17, 0.43 ± 0.18, 0.5 ± 0.15, 0.43 ± 0.18, and 0.36 ± 0.20, respectively (Supplementary Table 2). These results indicate that spatio-temporal patterns of spikes during both memory encoding and memory retrieval periods contain image category information that can be decoded by the classification model.

In both control cases, the model yields near-zero MCCs. The mean MCCs of the “Time-Shifted” case (Fig. 2c, bottom-left) for the five categories are 0.06 ± 0.11, 0.04 ± 0.09, 0.03 ± 0.10, 0.04 ± 0.11, and 0.03 ± 0.10, respectively (Supplementary table 3). The mean MCCs of the “Label-Shuffled” case (Fig. 2c, bottom-right) are all 0. These results affirm that the classification model effectively avoids overfitting and decodes real memory-related information from the hippocampal spiking activities.

### Spatio-Temporal Distribution of Category Information in Hippocampal Spikes

**Figure 3:**
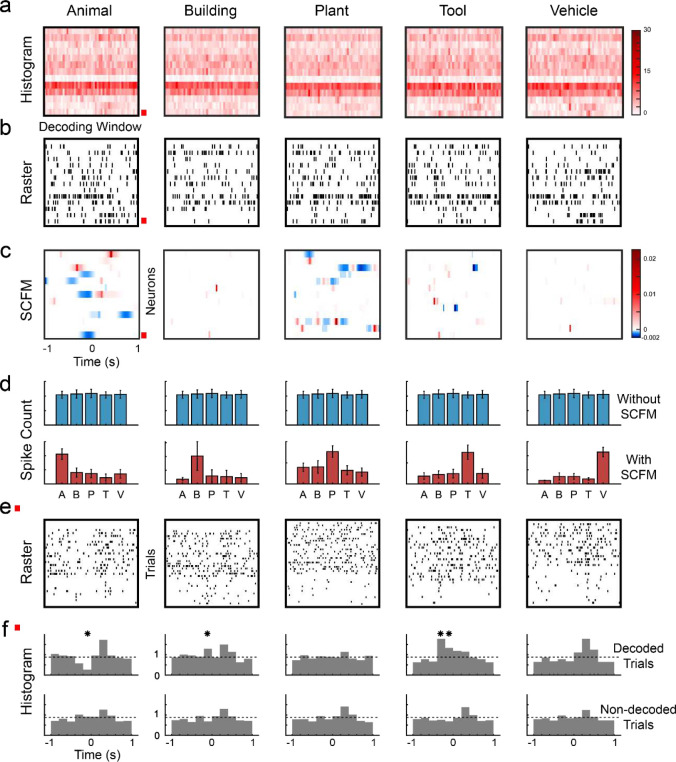
Spatio-temporal distributions of category information in hippocampal spike patterns are revealed by the classification model. **a**: peri-event histogram of spike patterns of the five categories during the Sample Response events. **b**: raster plots showing a single trial of the spatio-temporal patterns of spikes during the Sample Response event. The five categories cannot be easily distinguished in either **a** or **b**. **c**: SCFMs of the five categories in the classification model. The red box marks the neuron shown in **e** and **f**. Based on the SCFMs, this neuron contributes to encoding the five categories. **d**: spike counts of each category with (top panel) and without (bottom panel) using SCFMs as masks. SCFMs reveal the spatio-temporal regions of the spike patterns that encode the category information. **e**: spike raster plots of trials within each category of the neuron marked in **a**, **b**, and **c**. **f**: peri-event histograms of trials of the decoded category (top panel) and other (non-decoded) categories (bottom panel) of this neuron. Dashed lines represent the baseline firing rates. Significant differences in firing rates between decoded categories and non-decoded categories exist in time intervals consistent with the SCFMs (bins marked with asterisks, *p*<0.05).

The classification model used in this study is designed to be biologically interpretable^18^. It characterizes the mapping between input spikes to output categories by explicitly representing it in the form of sparse classification functional matrices (SCFMs) (Fig. 1), which quantifies spatio-temporal regions that maximize the differences between patterns of the decoded category and non-decoded patterns (i.e., patterns of other categories). The SCFM has the same dimension of the spatio-temporal pattern to be decoded. In an SCFM of a given category (Fig. 3c), zero-valued (white) areas do not contribute to the prediction of the memory category; positive (red) areas represent spatio-temporal regions where observing spikes increases the likelihood of the decoded category; negative (blue) areas represent regions where spikes decrease the likelihood of the decoded category.

A representative model is presented to illustrate how the model decodes the spatio-temporal patterns of spikes (Fig. 3). It is evident that the five categories cannot be easily distinguished from either the averaged spiking activities (Fig. 3a) or the single-trial spiking activities (Fig. 3b). There is no significant difference in the total spike counts within the patterns across the five categories (Fig. 3d, top). However, after applying the SCFMs of the five categories identified by the classification model (Fig. 3c) as masks to the spike patterns, i.e., calculating the inner product of the patterns and the SCFMs, these patterns show significant differences in spike counts within the SCFM regions across the five categories (Fig. 3d, bottom). This result shows that the model identifies the spatio-temporal regions critical for encoding the five categories of visual memories and further decodes these categories at the neuronal population level.

Based on the model, neurons with non-zero values in their SCFM contribute to the encoding of categories. This can be verified at the single-neuron level by comparing the firing patterns of the trials of the decoded category and non-decoded categories of these neurons (Fig. 3e). As shown in one example neuron, there are significant differences in firing rates between decoded categories and non-decoded categories in time intervals consistent with the non-zero regions of the SCFM (Fig. 3f).

### Sparseness of Spatio-Temporal Encoding of Categories

Encodings of the five categories are further characterized regarding spatial and temporal sparseness using classification models of all subjects (n=24). Spatial sparseness refers to the proportion of neurons with only zero values in the SCFMs (i.e., no contribution to encoding) relative to the total number of neurons in each neuron ensemble. Temporal sparseness represents the proportion of zero-valued time intervals of each individual neuron within its decoding window.

**Figure 4:**
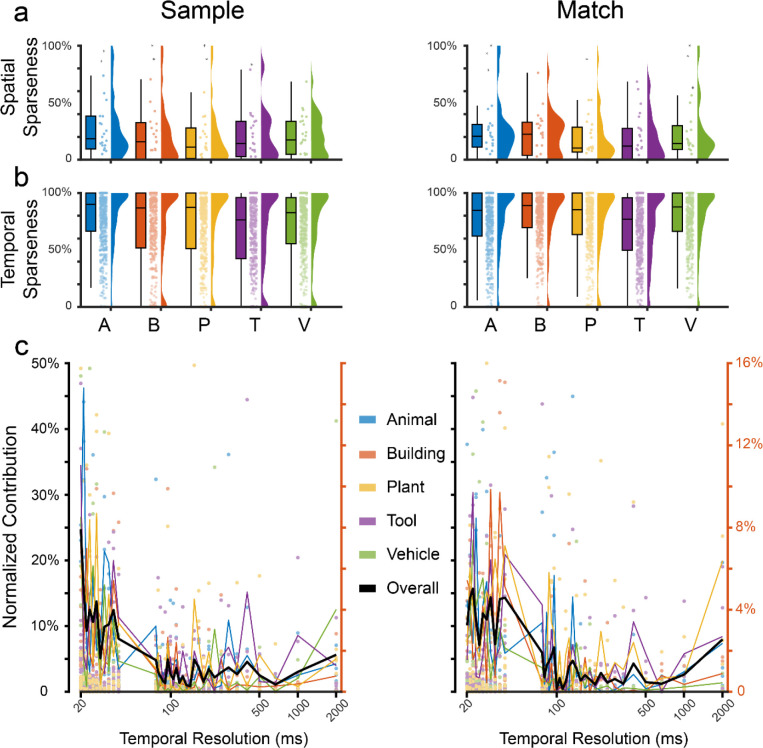
Sparseness of spatio-temporal encoding of visual memory categories during Sample Response and Match Response events of the DMS task. **a**: spatial sparseness of all neuron ensembles (n=24). **b**: temporal sparseness of all neurons (n=721). Bars: mean sparseness; error bars: standard deviation (STD) of sparseness. **c**: contribution of temporal resolutions to the encoding of visual memory categories. Colored dots (left y-axis): contribution of temporal resolutions to the encoding of categories in each subject. Colored lines (right y-axis): averaged contribution of temporal resolutions across all subjects (n=24). Black line (right y-axis): averaged contribution of temporal resolutions across all five categories. Left: Sample Response; Right: Match Response.

Results show that hippocampus neurons encode the five categories with similar levels of spatial and temporal sparseness (Fig. 4a, b). During the Sample Response, the spatial sparseness of the five categories is 28.2 ± 27.0%, 24.1 ± 28.3%, 21.1 ± 27.2%, 21.5 ± 23.1%, and 20.1 ± 18.3%, respectively. The temporal sparseness of the five memory categories is 78.8 ± 26.1%, 69.8 ± 35.3%, 71.5 ± 33.4%, 65.2 ± 34.9%, and 69.6 ± 33.5%, respectively. During the Match Response, the spatial sparseness of the five categories is 28.1 ± 27.3%, 21.8 ± 18.7%, 19.2 ± 21.5%, 18.9 ± 20.2%, and 24.9 ± 27.6%. The temporal sparseness of the five memory categories is 75.8 ± 28.1%, 77.9 ± 28.0%, 74.4 ± 30.0%, 69.8 ± 30.2%, and 76.9 ± 28.2%. These results indicate that hippocampal neuron ensembles encode visual memory categories in a distributed manner (population coding), i.e., with lower spatial sparseness, while individual hippocampal neurons encode visual memory categories with temporal codes, i.e., with high temporal sparseness.

### Temporal Coding of Visual Memory Categories

The ensemble multi-temporal-resolution model incorporates a large range of temporal resolutions in its base-learners, and the meta-learner further chooses the optimal subset of base learners to decode the memory categories (see “Methods”). Using a permutation feature importance (PFI) analysis^19^ on the base learner of the model, we directly quantify the contribution of each temporal resolution to the encoding of visual memory categories by calculating the reduction of the normalized cross-entropy loss^20^ caused by permuting the features associated with each temporal resolution.

Results show that a wide range of temporal resolutions are utilized in encoding the five categories, with high temporal resolutions playing a more important role than low temporal resolutions (Fig. 4c). Similar distributions of different temporal resolutions’ contributions are observed in all five categories during both the Sample Response and Match Response events of the DMS task. These results are consistent with the temporal sparseness results above and strongly suggest a temporal code, as opposed to a rate code, of hippocampal neurons in encoding visual memory categories.

### Contribution of Hippocampal CA3 and CA1 Neurons to Encoding of Categories

**Figure 5:**
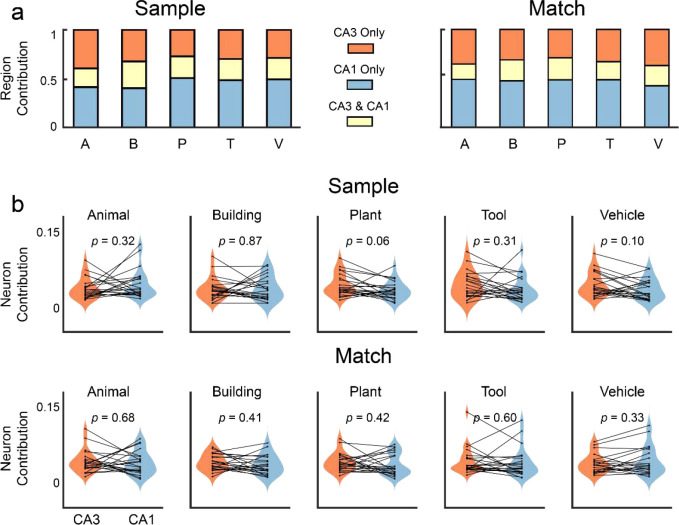
Contribution of hippocampal CA3 and CA1 to the encoding of visual memory categories during Sample Response and Match Response events of the DMS task. **a:** Contributions of the CA3 and CA1 regions. Red: unique contribution of the CA3 region; Yellow: redundant contribution shared by CA3 and CA1 regions; Blue: unique contribution of the CA1 region. **b:** averaged contribution of hippocampal CA3 and CA1 neurons to the encoding. Each dot represents one subject (n=24). There is no significant difference between CA3 and CA1 neurons during both Sample Response and Match Response (paired *t*-test).

In this study, we recorded neural spiking activities from hippocampal CA3 and CA1 regions (see “Methods”). To investigate how CA3 and CA1 neurons contribute to the encoding of memory categories, we further apply the PFI analysis to calculate the contribution of these neurons using their corresponding model features. First, the total contribution of each region, i.e., CA3 and CA1, is calculated as the reduction of the normalized cross-entropy loss by permuting all features associated with all neurons in that region. Redundancy is calculated as the contribution shared by the two regions, i.e., the summation of the individual contributions of the two regions subtracts the total contribution of the two regions combined. The unique contribution of each region is then calculated as the individual contribution of each region, subtracting the redundancy. Results show that CA3 and CA1 neurons contribute similarly to encoding the five visual memory categories with a significant amount of redundancy (Fig. 5a, b). The unique contribution of CA3, the redundancy, and the unique contribution of CA1 averaged across all five categories are 31.5 ± 4.9%, 22.5 ± 3.0%, and 46.0 ± 4.9% in the Sample phase, and 33.0 ± 3.2%, 19.8 ± 2.7%, and 47.2 ± 2.7% in the Match phase.

To balance the unequal numbers of CA3 and CA1 neurons in each subject, the averaged contributions of individual CA3 and CA1 neurons are calculated by normalizing the overall contributions of CA3 and CA1 ensembles to encoding with the number of CA3 and CA1 neurons in each region. Results show no significant difference between CA3 and CA1 neurons in all five categories during both the Sample Response and Match Response events of the task (Fig. 5c, d). These results indicate that CA3 and CA1 neurons contain similar information about visual memory categories, possibly due to the strong and divergent synaptic connection from CA3 to CA1 regions^17^.

## Discussion

This study combines human electrophysiology and computational modeling to investigate how the hippocampus encodes visual memory categories with its spiking activities.

The first main finding of this study is that visual memory categories can be successfully decoded from hippocampal CA3 and CA1 spikes during both the Sample (encoding) and Match (retrieval) phases of the DMS task. This result confirms the pivotal role of the hippocampus in integrating sensory information, such as the “what” information of objects, for the formation of episodic memories. It strongly suggests that, due to the very high dimensionality of the object space, the hippocampus uses categories to reduce the dimensionality and parsimoniously encode object information using its neuronal ensembles.

In addition, we demonstrate that memory categories can be decoded from single trials of hippocampal ensemble spike patterns using a classification model, while the traditional decoding methods often rely on averaged neuronal firing patterns across many trials^12–14^. This was made possible by the modern machine-learning techniques used in this study. Formulated as a classification problem, spatio-temporal patterns of the neuronal ensemble are used as the input signals, which allows all neurons to contribute collectively to the decoding of output signals (i.e., memory categories). In this model, sparse classifiers and ensemble learning techniques, such as bagging and stacking^21,22^, are used to effectively avoid overfitting and enable decoding memory categories from very high-dimensional input signals using relatively short data lengths. Importantly, this classification does not rely on a predefined temporal resolution in decoding. Instead, it incorporates a broad range of temporal resolutions in its base learners to extract multiscale temporal features from spike patterns. The optimal temporal resolutions are then determined using a data-driven stacking method by the meta-learner. This approach enables us to quantify the temporal resolution of decoding and address the key questions of which coding strategy, temporal or rate coding, is employed in the hippocampus.

In contrast to many machine learning models that operate as “black boxes,” the classification model used in this study is highly interpretable. It offers intuitive representations of the spatio-temporal characteristics of spike patterns, in the form of SCFMs, that are most relevant for decoding. This interpretability has been rigorously validated through both simulated data and experimental results from rodent studies^18^, enabling further meta-analysis of the SCFMs to gain additional insights into hippocampal memory encoding.

Through meta-analyses of the SCFMs, we found that the hippocampus encodes memory categories in a spatially distributed and temporally sparse manner. Across all five categories, 70–80% of neurons are involved in encoding. However, within each neuron, only 20–30% of the temporal window contributes to encoding these categories. This result is consistent with previous human studies on episodic memories using non-visual stimuli such as words^16^. From a computational modeling perspective, such an encoding strategy is almost inevitable since it is efficient in balancing between maximizing the capacity for storing diverse memories and minimizing the energy (which is positively related to the number of spikes) required to encode each memory^23^.

Additionally, this study offers new insights into the long-standing debate about the relative importance of rate coding versus temporal coding strategies used by neurons to represent information, including memory^2,11,15,24–27^. Although hippocampal neurons utilize both strategies^15,26^, our findings indicate that temporal resolutions play a more significant role in encoding visual memory categories. This is also consistent with the temporal sparseness findings and suggests a temporal code, as opposed to a rate code, of hippocampal neurons in encoding visual memory categories.

Moreover, we found that neurons in both the hippocampal CA3 and CA1 regions contribute similarly and contain redundant information about memory categories. This is expected given the strong and diffuse synaptic projections from CA3 pyramidal neurons to CA1 pyramidal neurons^17^. As a result, information in the CA3 region likely mirrors that in the CA1 region. In our previous studies on developing hippocampal memory prostheses, we demonstrated that the spiking activity of CA1 neurons can be accurately predicted from CA3 neuron activity using a multi-input, multi-output (MIMO) machine learning model^28–30^. The presence of similar information in both regions enables the success of such a model, even when utilizing nonlinear dynamical models capable of capturing complex input-output transformations.

Several limitations of this study should be acknowledged, along with opportunities for future research. Due to constraints in clinical studies, we used a relatively small set of images and only five largely independent categories. In everyday life, humans encounter vast numbers of diverse objects belonging to numerous correlated or uncorrelated categories. To fully investigate memory encoding in a more naturalistic context, advanced recording techniques and modeling methods will be required. Additionally, in this study, the classification model was primarily used as a tool to explore how memory categories are encoded in spatio-temporal spike patterns. However, because this model maps memory content to neural activity, it also has potential applications in facilitating memory encoding through model-based stimulation patterns, which could be valuable in the development of hippocampal memory prostheses aimed at restoring or enhancing memory functions^31–33^.

## Methods

### Human Hippocampal Recording

**Figure 6.**
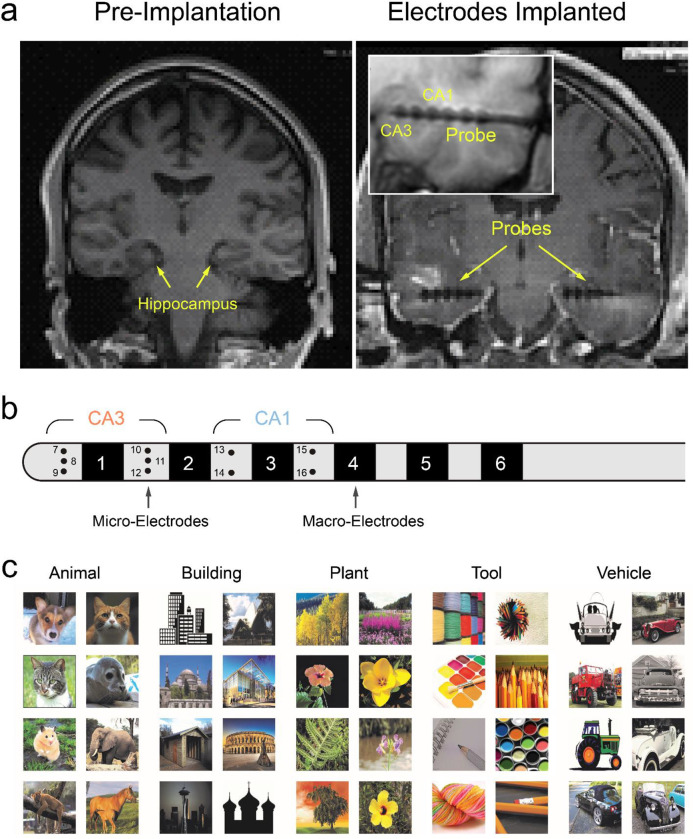
Human experimental paradigm. **a**: 3T MRI showing pre-implantation hippocampal structures and post-implantation electrode locations in one subject. Inset: zoomed-in view of the probe in the hippocampus. **b:** Layout of the micro-macro probe containing 6 macro-electrodes and 10 micro-electrodes. Six and four micro-electrodes were implanted in the CA3 and CA1 regions, respectively. **c**: Sample Images of the five memory categories used in the DMS task.

All subjects enrolled in this study were diagnosed with refractory focal epilepsy and underwent intracranial depth electrode implantation for seizure localization and monitoring (Fig. 6a). Typically, each subject received 1–4 FDA-approved Ad-Tech (Medical Instrumentation Corporation or PMT Corporation) “Macro-Micro” depth electrodes. The “macro”-electrodes recorded low-frequency signals such as clinical electroencephalography (EEG), while the “micro”-electrodes recorded higher-frequency signals like single-unit activity (spikes). Throughout the experiment, brain signals were recorded using the Blackrock Cervello system concurrently with a clinical EEG recording system. Electrode placement was performed intraoperatively using either a stereotactic headframe or a frameless stereotactic system, with the aim of aligning the electrodes perpendicularly to the long axis of the hippocampus, targeting both CA3 and CA1 regions. Post-operative MRI and electrophysiological recordings confirmed the accuracy of the electrode placements (Fig. 6a). Neural activities were monitored intraoperatively during electrode placement to confirm local field potentials and spikes recordings. In this study, each probe contained 10 micro-electrodes, with 6 in the CA3 region and 4 in the CA1 region (Fig. 6b). Spikes were obtained by isolating single-unit action potential waveforms from continuous recordings through online (Blackrock Cervello system) and offline (Plexon Offline Sorter) spike sorting procedures.

All procedures were reviewed and approved by the Institutional Review Board of the University of Southern California and Wake Forest University in accordance with the National Institute of Health. All subjects provided voluntary written informed consent prior to participation in this study. Experiments were performed at Keck Hospital of the University of Southern California (Keck), Rancho Los Amigos Rehabilitation Center (Ranco), and Wake Forest Baptist Medical Center (Wake).

### Behavioral Task

Subjects were given a recovery period of 1–2 days from the anesthesia. Patients remained in the Intensive Care Unit (ICU) of Keck and Rancho and the Epilepsy Monitoring Unit (EMU) of Wake during the duration of their time in the hospital, where all behavioral tasks were performed. Subjects performed the memory-dependent DMS task with a touch-screen computer while sitting either in a bed or in a chair next to the bed in the ICU/EMU. Each DMS trial commenced with the display of a focus ring in the center of a touch screen (Fig. 2a). Subjects were instructed to click on the focus ring to initiate the Sample phase. In the Sample phase, a sample image was presented at a randomly selected location on the touch screen (Sample Presentation event). Subjects were instructed to remember this sample image and subsequently click on it to trigger a Sample Response event. Upon clicking, the sample image disappeared, and the screen remained blank for 3–5 seconds (Delay Phase). Following the Delay Phase, multiple images, including the sample image, were simultaneously presented on the touch screen at different locations. Subjects were instructed to select and click on the sample image based on their memories to generate a correct Match Response. One DMS task session consisted of 100–150 trials. Each subject completed 1–2 sessions of the DMS task.

Sample images (n=500) were obtained from the internet. Five main categories (“Animal”, “Building”, “Plant”, “Tool”, and “Vehicle”) of images were included in the DMS task (Fig. 6c). Due to the diversity within the “Tool” category, images in this group were limited to handicraft consumable items such as pencils, pens, markers, crayons, thread, yarn, and similar objects. Volunteers labeled the categories (1: in category, 0: not in category) of these images using an online survey^28^. Each image was labeled 56 times on average. Only images with high scores (>0.9) in their respective categories were included. Binary labels of categories of images were used as the output signal of the memory decoding model.

### Memory Decoding Model

The memory decoding model decodes spatio-temporal patterns of spikes into binary visual memory category labels (Fig. 1). It consists of two layers of learners (classifiers).

In its first layer, a bank of base learners (*L*1-regularized logistic regression classifiers)^34^ extracts spatio-temporal features from spike patterns with a wide range of temporal resolutions using B-spline functions with different numbers of knots^33,35^. Each base-learner, using a single temporal resolution, acts as a weak classifier on its own. *L*1-regularization is used to reduce feature dimensionality and yield sparse estimation of model coefficients^34^. Bagging method is adopted to reduce estimation variances by partitioning the data into multiple replicas and estimating multiple copies of the base learners with these replicas (ensemble classifier)^21^.

In its second layer, a meta-learner combines outputs from the base learners, each operating at a single temporal resolution, into an ensemble model using another *L*1-regularized logistic regression classifier. It fuses multiple temporal resolutions into the model to classify spatio-temporal patterns of spikes into memory category labels. It renders the model multi-temporal resolution and a stronger classifier. The model can be mathematically expressed as

$$y=g(f_{r}^{\left(m\right)}(X))$$

where x is the input spike pattern, y is the output memory label, f(⋅) is the base-learner, g(⋅) is the meta-learner, m is the number of B-spline knots controlling the temporal resolutions, r is the index of the bagging replica. Given a set of B-spline knots, the temporal resolution of each base-learner is M/(m+1), where M is the length of decoding window (2 seconds in this study). More methodological details of this memory decoding model can be found in Supplementary Material 2 and our previous publication^18^.

Nested cross-validation is applied throughout the estimations to prevent overfitting^36,37^. Model coefficients are estimated using training data. Hyperparameters are optimized using validation data. Model performance is evaluated with test data that are held out from training and validation data.

This model has been extensively evaluated using both synthetic and rodent data^18^. Specifically, synthetic data results indicate that the model could identify optimal temporal resolutions by appropriately assigning weights to base learners with different resolutions. It can faithfully recover the ground truth temporal resolutions and firing probability intensity functions of the model neurons. When applied to the hippocampal spiking data recorded from rats performing a memory-dependent delayed nonmatch-to-sample task, the model highly accurately decodes spatial memory information.

One important advantage of this model is its interpretability. It generates SCFMs representing the spatio-temporal characteristics of hippocampal spike patterns most relevant to classification. SCFMs are calculated by nonlinearly integrating the predictions of the base-learners using the meta-learners^18^ as

$$F^{\prime }(n,\tau )={\left\{1+exp(-{w_{0}}^{\prime }-\sum_{m=1}^{Q}\left[\sum_{j=1}^{J}b_{j}^{m}(\tau )w(n,j)\right]w^{\prime }(m))\right\}}^{-1}$$

where $b_{j}^{m}(\tau )$ are the B-spline basis function of each base-learner; w are the model coefficients of base learners f(⋅); J is the total number of B-spline knots used in a specific base-learner. $w^{\prime }$ are the model coefficients of the meta-learner g(⋅). Q is the total number of base learners. More methodological details of the SCFM can be found in Supplementary Materials 2 and our previous publication^18^.

When decoding a specific memory category, having spikes in SCFM regions with positive values increases the probability of the pattern belonging to the decoded category. By contrast, having spikes in negative regions of the SCFM decreases this probability. This allows for a direct quantification of specific spatio-temporal regions that maximize the differences between patterns of the decoded category and non-decoded patterns.

Model performance is assessed using the Matthews correlation coefficient (MCC), which effectively handles imbalanced data. The MCC is calculated from the confusion matrix components, i.e., true positives (TP), true negatives (TN), false positives (FP), and false negatives (FN), using the formula

$$MCC=\frac{TP\;\times \;TN\;-\;FP\;\times \;FN}{\sqrt{\left(TP\;+\;FP)\;\times \;(TP\;+\;FN)\;\times \;(TN\;+\;FP)\;\times \;(TN\;+\;FN\right)}}$$

This coefficient provides a value between −1 and 1, where −1, 0, and 1 indicate opposite, random, and perfect classification, respectively. Models that predict all outputs as a single class (i.e., all 1 or all 0), or randomly (chance level) will result in an MCC of 0.

Permutation feature importance (PFI) analysis quantifies the contribution of individual or groups of features to the predictive power of a model by evaluating the impact of their random alteration^19,37^. The process involves permuting each input feature or each group of input features independently and observing the resultant increase in model loss (e.g., cross-entropy in this study). This increase is a direct indicator of the feature’s importance; a significant rise in model loss suggests a high dependency of the model on that feature for accurate predictions. Thus, the importance of each feature is assessed based on the degree to which randomizing the feature degrades model performance.

## Data Availability

Both raw data and processed data used in this study are available from the corresponding author on request. Code and tools for the memory decoding model are available at https://github.com/neural-modeling-and-interface-lab/Double-Layer-Multi-Resolution-Memory-Decoding-Model.
